# Histopathology and molecular pathology confirmed a diagnosis of atypical Caroli’s syndrome: a case report

**DOI:** 10.1186/s13000-024-01462-9

**Published:** 2024-02-22

**Authors:** Tianmin Zhou, Keyu Liu, Hao Wei, Qingmei Zhong, Daya Luo, Wenjuan Yang, Ping Zhang, Yingqun Xiao

**Affiliations:** 1 Department of Pathology, Infectious Diseases Hospital of Nanchang University, Nanchang, 330001 Jiangxi China; 2 Queen Mary School, Nanchang University, Nanchang, 330006 China; 3 The First Clinical Department, Nanchang University, Nanchang, 330006 China; 4 Department of Biochemistry and Molecular Biology, School of Basic Medical Sciences, Nanchang University, Nanchang, 330006 China; 5 Infectious Diseases Hospital of Nanchang University, Nanchang, 330001 Jiangxi China

**Keywords:** Caroli’s syndrome, Case report, Histopathology, Molecular Pathology, PKHD1

## Abstract

Caroli’s syndrome is a congenital disease characterized by dilation of intrahepatic bile ducts and congenital hepatic fibrosis. It is a rare condition in clinical work. Typically, the diagnosis of this disease is confirmed through medical imaging. Here, we report a case of atypical Caroli’s syndrome in a patient who presented with recurrent upper gastrointestinal tract bleeding. The patient underwent imaging examinations, liver biopsy and whole exome sequencing. The results of the imaging examination were non-specific. However, with the aid of pathological examination, the patient was diagnosed with Caroli’s syndrome. In conclusion, for cases where the imaging presentation of Caroli’s syndrome is inconclusive, an accurate diagnosis should rely on pathology. By discussing this specific case, our aim is to enhance readers' understanding of this disease, provide valuable information that can aid in the early detection and appropriate management of Caroli’s syndrome, ultimately improving patient outcomes.

## Introduction

Caroli’s disease (CD) is a rare congenital disease characterized by segmental saccular dilation of intrahepatic bile ducts without obstruction. It was first reported by Caroli et al. in 1958 [1]. The incidence of CD is approximately 1 in 1,000,000 live births [2]. It is widely recognized that CD is associated with mutations in the polycystic kidney and hepatic diseases 1 (PKHD1) gene [3, 4]. According to the diagnostic criteria for Caroli’s disease, it is classified into two subtypes: Caroli’s disease type I, characterized by cystic segmental dilation of the bile ducts, and Caroli’s disease type II or Caroli’s syndrome (CS), which is associated with saccular alterations of the hepatic ducts, liver fibrosis, and even cirrhosis with manifestations of portal hypertension [5], as observed in our patient. CS is typically diagnosed during adolescence or before the age of 30, often coinciding with the time when patients seek medical attention due to apparent symptoms [6, 7]. The diagnosis of typical CS is not challenging, as it can be identified through clinical symptoms and imaging techniques such as abdominal ultrasound, computed tomography (CT), and magnetic resonance imaging (MRI). Most cases of typical CS are associated with autosomal recessive polycystic kidney disease (ARPKD), which often presents with varying degrees of renal cysts. In addition, patients with unexplained hepatic fibrosis and portal hypertension may exhibit non-obstructive segmental or diffuse cystic dilation of the intrahepatic bile ducts. The most characteristic imaging finding is the “central dot sign” [8], although the presence of fibrosis in CS can sometimes obscure this sign on radiological examinations. However, the diagnosis of atypical CS can be challenging due to the absence of characteristic clinical manifestations and imaging features. In this case report, we describe the case of a young male patient with atypical CS who does not have ARPKD, polycystic liver, or the characteristic “central dot sign”. The patient's only presenting symptom is unexplained liver cirrhosis.

## Case presentation

### General conditions and clinical manifestations

The 17-year-old male patient has been experiencing recurrent fatigue for 8 years and melena for the past 5 days. He has received treatment at a local hospital on multiple occasions, where a Color Doppler Ultrasound indicated liver cirrhosis and splenomegaly. He was subsequently diagnosed with liver cirrhosis following hepatitis and was treated with hemostatic drugs and fluid supplementation. However, the treatment at the local hospital failed to alleviate the symptoms of anemia. As a result, the patient sought new diagnosis and treatment at our hospital.

Upon examination, the patient's general condition was stable. The pale face observed was consistent with anemia, while no obvious signs of jaundice or Kayser-Fleischer rings were identified in either eye. Additionally, there were no signs of liver palm or spider angioma. Palpation did not reveal any superficial lymph node enlargement, abdominal mass, liver enlargement, pressing pain, or positive Blumberg sign. A firm and obtuse spleen was palpated 6 cm below the anterior rib margin using two-handed palpation. Abdominal shifting dullness was negative.

The patient denied a history of HBV infection, alcohol consumption, smoking, or exposure to schistosomiasis epidemic areas. Both parents are healthy, although the patient's younger sister and paternal grandmother have a history of liver cirrhosis.

### Laboratory examination

The blood and biological tests of this patient revealed elevated levels of total bilirubin (TBiL) and serum amyloid A (SAA), as well as depressed levels of prealbumin and high-density lipoprotein (HDL). The results of hepatic enzymes were within the normal range. These findings suggest the presence of cholestasis, inflammatory reaction, and liver dysfunction.

### Imaging examination

**Fig. 1:**
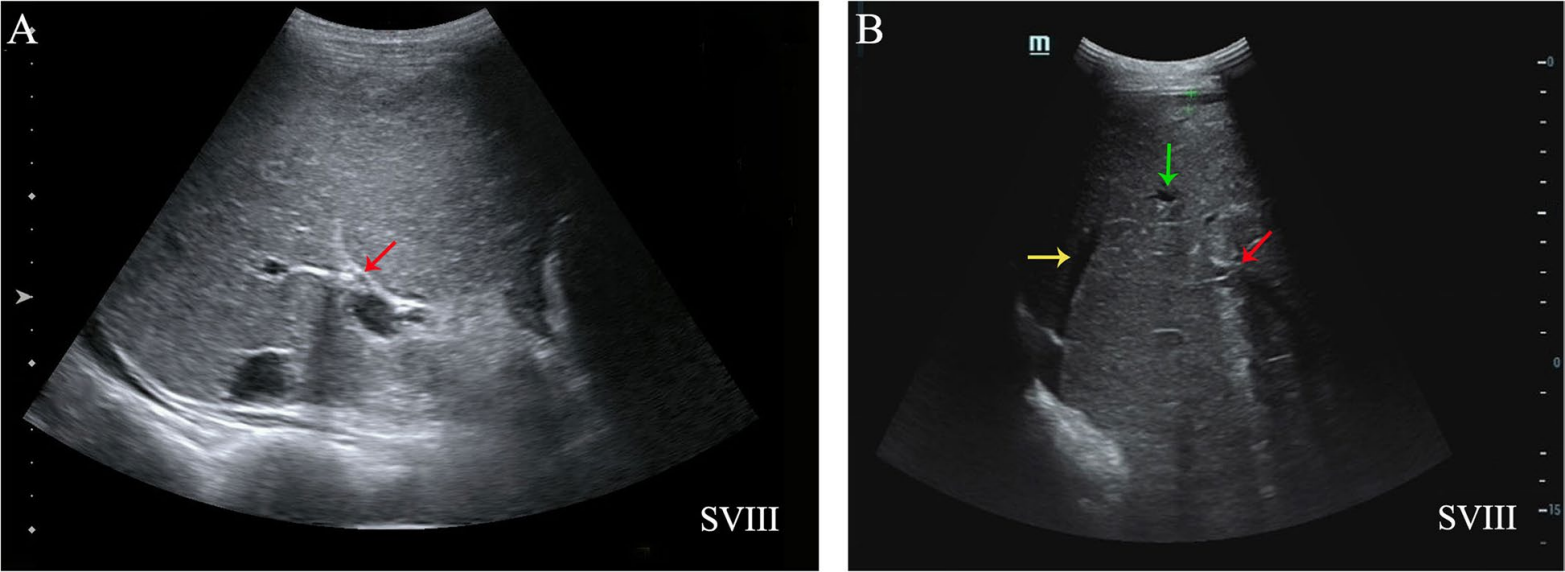
USG Abdomen reveals two examinations conducted at different time prior to the current observation (**A** and **B**). Right portal vein (red arrowhead); Right hepatic vein (yellow arrowhead); the third-order biliary branches (green arrowhead); Right anterior lobe of the liver (SVIII)

Abdominal B-mode ultrasound showed the oblique diameter of the right lobe of liver is 11.4 cm (normal 10–14 cm); the third-order biliary branches measuring 0.59 mm (normal < 0.4 mm). Glisson’s capsule was coarse. Echoes of hepatic area were uneven, and hepatic veins were not clear. The diameter of portal vein and common bile duct caliber were 13 mm (normally < 13 mm) and 4 mm (normally 6–8 mm) respectively [9]. No obvious of polycystic liver was observed (Fig. 1).

**Fig. 2:**
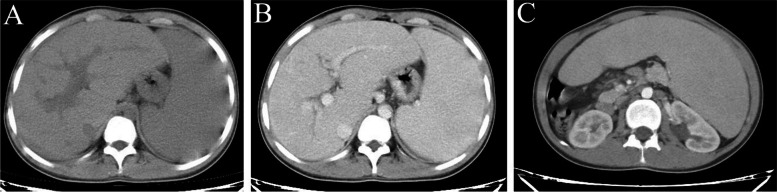
The results of CT scan. **A** Plane CT scan; **B** Delayed phase of contrast-enhanced CT; **C** CT scan shows giant spleen and normal kidneys

Abdominal computer tomography (CT) measured the maximum liver diameters and showed the cranio-caudal (CC) was 15.0 cm (normal < 15 cm), anterior–posterior (AP) of right and left lob were 13.4 cm (normal 8–10 cm) and 9.3 cm (normal 6–9 cm) [10]. Importantly, no abnormal highdensity shadow or intrahepatic cyst was observed. The volume of spleen was obviously enlarged, extending into the pelvis and across the midline. The kidneys exhibited a normal morphology without evidence of polycystic kidney disease (Fig. 2).

**Fig. 3:**
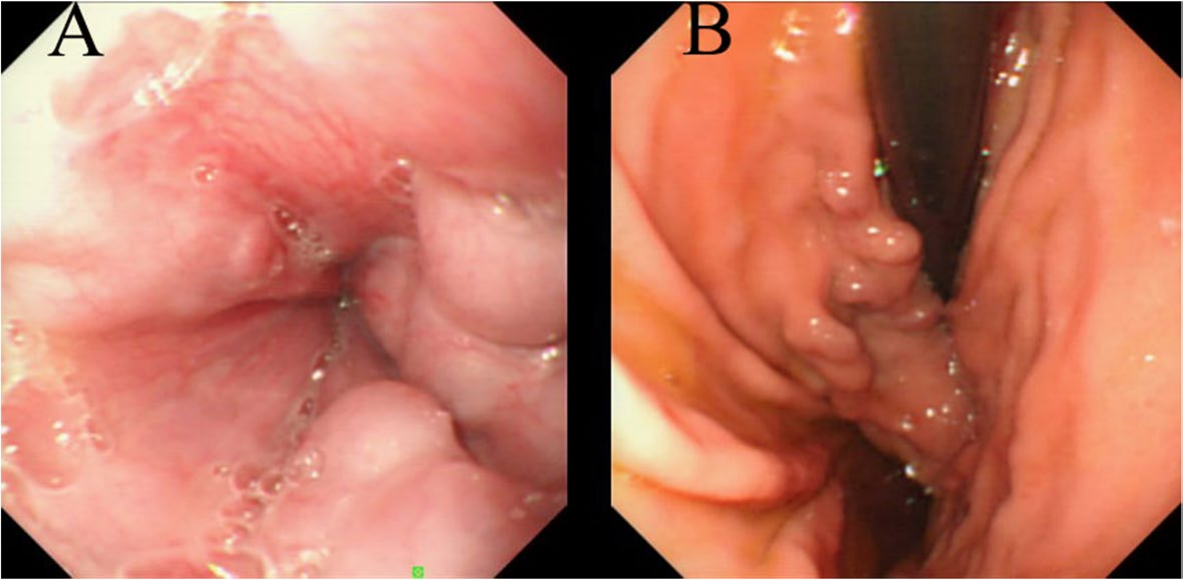
Gastric endoscopy of Caroli’s disease shows that (**A**) 4 variceal veins in esophageal wall; **B** several variceal veins extending from cardia, RC +

Under gastric endoscopy, it clearly showed that there were several circuitous bulges protruding into the cavity of esophagus and gastric fundus. The results showed severe esophagogastric varices. The diameter of fundus varices was about 6 mm. Red-color sign in fundus varices was positive (Fig. 3).

### Pathological examination

**Fig. 4:**
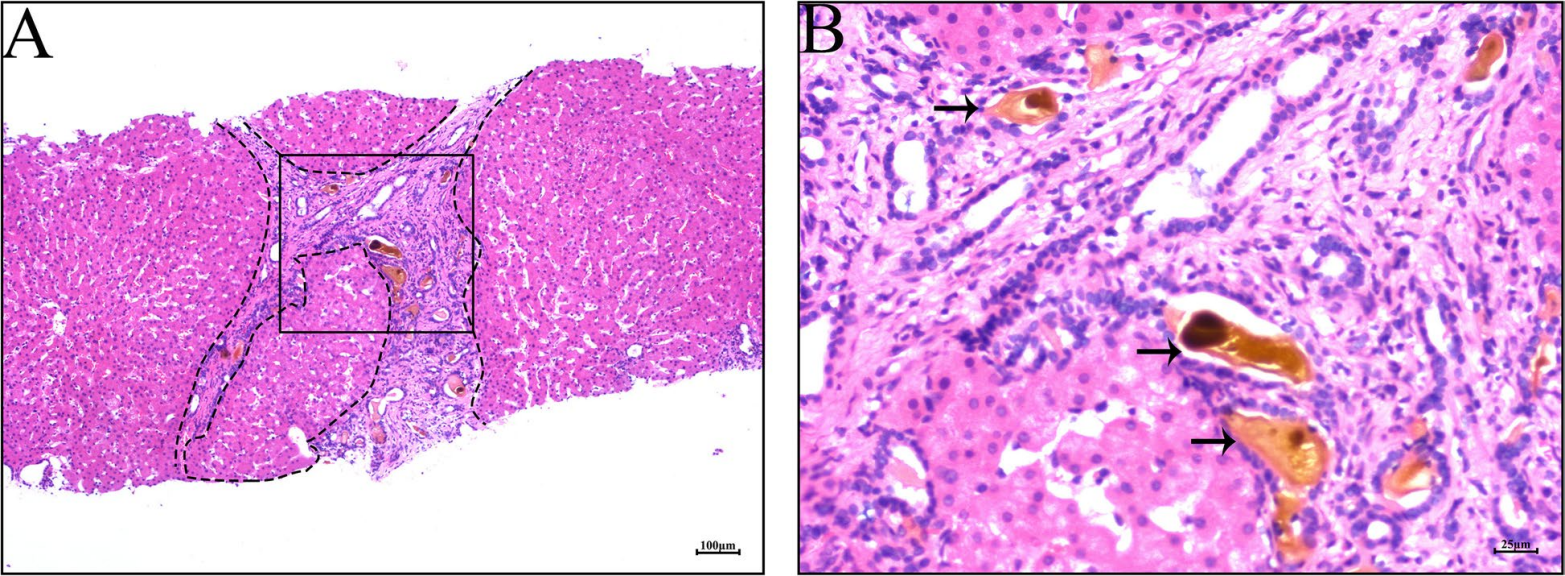
**A** Hematoxylin–eosin (H-E) stain (× 50) shows severe portal tracts fibrotic bands and “pseudolobules” (shows in black dashed circle); **B** HE stain (× 200): Bile ductal malformation, dilation and cholestasis (black arrowhead); The (**B**) is enlarged in black box of (**A**)

Histological examination of liver biopsies stained with Hematoxylin–eosin (HE) revealed distinct features of the liver tissue. The portal tracts showed significant expansion of severe fibrosis, with the formation of bridging fibrosis. Infiltration of inflammatory cells and the presence of pseudolobules were observed (Fig. 4A). Importantly, these “pseudolobules” differed from those typically associated with liver cirrhosis. The arrangement of hepatocyte plates appeared normal, without any indications of hepatocyte edema, degeneration, or cholestasis. This finding is consistent with the pathological features of congenital hepatic fibrosis reported by Ru-JiaT et al. [11]. Additionally, a large number of bile ducts within the portal tracts showed malformation and dilation, accompanied by hyperplastic cholangiocytes that showed no atypia. Notably, the dilated bile ducts showed numerous instances of cholestasis, as indicated by the presence of brown staining (Fig. 4B).

**Fig. 5:**
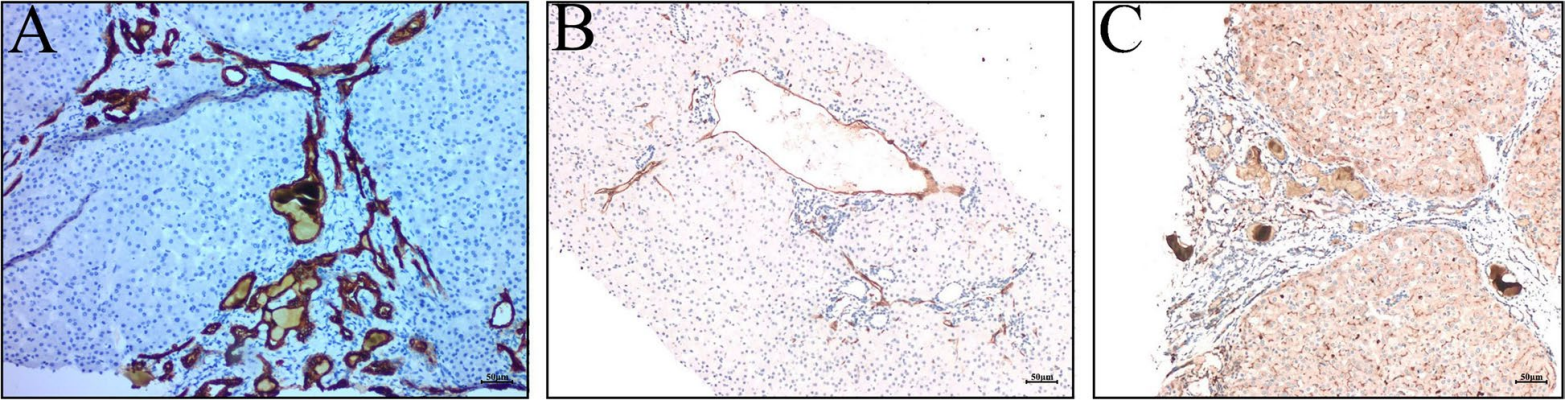
**A** anti-CK7 (× 100): biliary dilation and hyperplasia; **B** anti-CD34 (× 100): The interlobular vein in the portal area dilated significantly and protruded into the hepatic parenchyma, and the interlobular vein almost occupied the whole portal area; **C** anti-CEA (× 100): normal bile canaliculi inside of hepatic lobules

The use of immunohistochemistry and many special stains of tissue specimen can help us learn more about the pathological and functional changing, contributing to make a proper diagnosis. Anti-CK7 is the antibodies used to label cholangiocytes. The immunohistochemistry of CK7 clearly showed biliary dilation and cholangiocytes hyperplasia (Fig. 5A). In the hepatic lobule, anti-CD34 normally does not expressed in sinus endothelial cells inside of hepatic lobules except for neoangiogenesis and changes of blood composition [12, 13]. The positive results of anti-CD34 (Fig. 5B) showed that the interlobular vein in the portal vein dilated obviously and protruded into the hepatic parenchyma, and the interlobular vein occupied almost the whole area of the portal vein, suggesting that there may be portal hypertension, which is consistent with the clinical manifestation of the patient. We showed a normal structure of bile capillaries in hepatic lobules with anti-CEA (Fig. 5C).

**Fig. 6:**
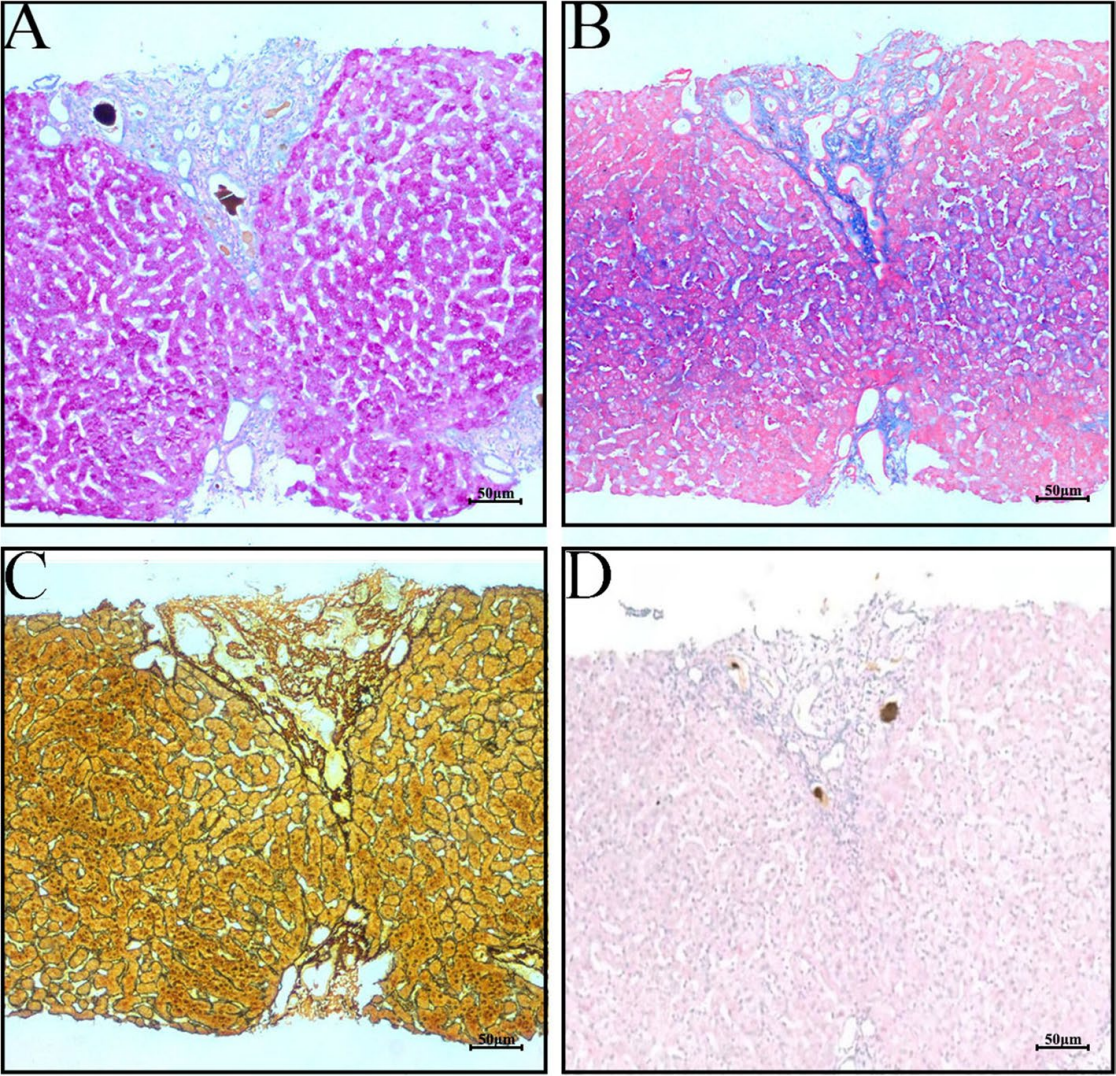
**A** PAS staining (× 100) and (**B**) Trichrome stain (× 100) shows peri-sinus and peri-portal fibrosis; **C** Reticular staining (× 100) shows portal fibrosis and “pseudolobule” formation; **D** D-PAS (× 100)

The liver biopsy specimens underwent special stains including periodic acid-Schiff (PAS) stain, Masson trichrome stain, reticulin connective tissue stain, and diastase pre-treated PAS (DPAS) stain. The PAS stain allows for the visualization of intracellular glycogen storage in hepatocytes, appearing as red color under microscopy. This stain is useful in diagnosing hereditary glycogen storage diseases or ischemic injuries [14]. In this study, the hepatocytes within the hepatic lobules exhibited red staining, indicating normal glycogen storage without intracellular fading or fatty vesicles. The Masson trichrome stain highlights collagen fibers in blue and provides information on the extent of fibrosis. The reticulin connective tissue stain is used to evaluate the degree of fibrosis by labeling reticulin (type III collagen fiber) deposition [15]. Both of these connective tissue stains revealed perisinusoidal and portal tracts fibrosis, bridging fibrosis, resulting in fibrotic septa and the formation of “pseudolobules”. The D-PAS stain is employed to identify phagocytic inclusions and immunoglobulin within macrophages. In this study, neither the portal tracts nor the liver parenchyma exhibited indications of acute phagocytosis by activated macrophages or immunoglobulin globules, which are associated with drug-induced hepatitis and autoimmune liver disease (Fig. 6).

Based on the observed interlobular bile duct malformation, dilation, cholestasis, and severe fibrosis in the patient’s clinical presentation, the diagnosis of CS was inferred in accordance with the 2022 EASL Clinical Practice Guidelines on the management of cystic liver diseases [16]. After extensive consultation with the patient, whole exome sequencing was performed to provide a thorough molecular analysis to confirm the diagnosis. This sequencing technique allowed for a detailed examination of the patient’s genetic profile, enabling the identification of potential disease-causing variants associated with CS and increasing the certainty of the diagnosis.

### Whole exome sequencing

Based on the pathological results, peripheral venous blood (5 mL) was collected from the patient, his affected sister, and his healthy parents for Whole Exome Sequencing (WES), which was performed by Macro & Micro-test. The sequencing results revealed two heterozygous variants: a missense variant c.2507 T > C (p.Val836Ala) and a novel variant c.10156G > C (p.Val3386Leu). Both the patient and the sister harbored these two variants, with the former being paternal and the latter being maternal (see Table 1).

**Table 1:** The results of whole exome sequencing

Family members	Gene	Position	Variants	RS/HGMD-ID	Hom/Het/Hemi*	ACMG grade
Patient	PKHD1 NM_170724 exon24	chr6:51910887	c.2507 T > C p.V836A	rs199568593	Het	Pathogenic/ likely pathogenic
	PKHD1 NM_170724 exon60	chr6:51609183	c.10156G > C p.V3386L	-	Het	Pathogenic/ likely pathogenic
Sister	PKHD1 NM_170724 exon24	chr6:51910887	c.2507 T > C p.V836A	rs199568593	Het	Pathogenic/ likely pathogenic
	PKHD1 NM_170724 exon60	chr6:51609183	c.10156G > C p.V3386L	-	Het	Pathogenic/ likely pathogenic
Father	PKHD1 NM_170724 exon24	chr6:51910887	c.2507 T > C p.V836A	rs199568593	Het	Pathogenic/ likely pathogenic
Mother	PKHD1 NM_170724 exon60	chr6:51609183	c.10156G > C p.V3386L	-	Het	Pathogenic/ likely pathogenic

## Treatment

The patient underwent various forms of treatment to address their symptoms. This included splenic artery embolization to restore splenic function and endoscopic esophageal varix ligation to stop bleeding in the upper gastrointestinal tract. In addition, the patient received acid suppression therapy and antibiotic treatment.

## Follow-up

Follow-up was performed by telephone interview for 2 years since the patient left hospital. No varices bleeding happened anymore. Vital signs were stable, and the patient has no discomfort.

## Discussion

Caroli’s syndrome (CS) is a rare congenital disease characterized by non-obstructive segmental saccular dilation of intrahepatic bile ducts [17]. It is often accompanied by congenital hepatic fibrosis (CHF), which presents as fibrosis in the portal tracts. In terms of pathogenesis, CS belongs to a disorder called ductal plate malformation (DPM), a congenital disorder caused by abnormal development of the embryonic biliary [18–20]. The diverse range of diseases within the DPM spectrum can present with unspecific clinical symptoms and signs, making diagnosis challenging for doctors [21, 22]. While several intracellular pathways may contribute to the pathogenesis of CD rats, the exact pathways in humans are still being researched [17]. The etiology of CS is complex and unclear. Some cases have reported a potential link between CS and PKHD1 gene mutation. It has been confirmed that PKHD1 mutation is the primary cause of autosomal recessive polycystic kidney disease (ARPKD) [23]. The PKHD1 gene encodes fibrocystin, which is expressed on renal epithelial cells and cholangiocytes [24]. The biological function of the PKHD1 gene is not fully understood, but it is believed to be involved in cystogenesis, tube morphogenesis, cell–cell junction, cilia function, planar cell polarity, and cell proliferation [24, 25]. The common clinical presentations of CS include upper abdominal pain, recurrent cholangitis, intrahepatic calculi, jaundice, undetermined cholestasis, portal hypertension, pancreatic damage, and spleen hyperfunction. Based on the literature, patients with CS could be easily misdiagnosed with many other hepatic diseases, such as primary sclerosing cholangitis (PSC), multidrug resistance protein 3 (MDR3) deficiency, recurrent pyogenic cholangitis, hepatic cysts, autosomal dominant polycystic liver disease (ADPKD), choledochal cysts, congenital hepatic fibrosis, biliary papillomatosis, and biliary hamartomas [26–28]. To make a diagnosis, doctors typically begin with imaging examinations when patients present with nonspecific clinical symptoms. When the patients with visible discomfort seek medical attention, imaging examination can usually reveal the presence of multiple dilated intrahepatic bile ducts. In most cases, imaging examinations can reveal multiple dilated intrahepatic bile ducts. The ‘central dot sign’ found on MRI and CT scan is highly specific for typical CS [29]. It is a high-dense dot in the dark background. Histologically, the dot is formed by portal veins and hepatic arteries, while the background consists of dilated intrahepatic bile ducts. However, this case does not exhibit this specific sign in the imaging examination results. Therefore, pathological examinations and gene sequencing were performed to confirm the diagnosis.

In this case report, the patient denied any history of HBV infection. The immunohistochemistry results for HBV surface antigen and core antigen were negative (figures not shown), indicating that acute or chronic hepatitis B was not present. Wilson's disease is a rare hereditary disease caused by copper accumulation, which can involve the liver and central nervous system. Liver symptoms, including jaundice, portal hypertension, and fibrosis, often occur before the age of 20, while neurological symptoms typically occur much later [30, 31]. No Kayser-Fleischer (K-F) rings were found in the eyes, and the patient’s psychiatric condition was normal. Blood tests showed a normal level of ceruloplasmin (Table 2). The likelihood of hereditary Wilson's disease was low, so we did not recommend sequencing the ATP7B gene. Blood tests also showed negative serum autoantibody. Liver biopsy revealed no lymphoplasmacytic infiltration into hepatic lobules, rosette formation in hepatocytes, or interface hepatitis [32]. These results ruled out the possibility of autoimmune hepatitis. Previously known as primary biliary cirrhosis, primary biliary cholangitis (PBC) is an autoimmune liver disease characterized by the destruction of interlobular bile ducts, cholestasis, portal tracts inflammation, and fibrosis [33]. The histological features of CS can be easily distinguished from those of PBC. PBC is histologically characterized by chronic non-suppurative destructive cholangitis of the small interlobular bile ducts, resulting in chronic progressive cholestasis. In this case, microscopic observation showed no obvious bile duct damage in the portal tracts; instead, bile duct hyperplasia and dilation were observed. This suggests that the diagnosis of PBC was excluded. The diagnosis of drug-induced liver injury (DILI) should be considered after excluding other possibilities. The results of PAS-D staining did not show clusters of macrophages, and the patient had no history of drug use. Therefore, the results did not meet the criteria for a diagnosis of drug-induced hepatitis. The results of PAS staining revealed normal glycogen storage in hepatocytes, and no abnormal uncolored hepatocytes were found, indicating no steatosis. The results of anti-CD34 staining in this case suggest the presence of portal hypertension, which is consistent with the patient's clinical manifestation. The final diagnosis was based on the patient's family history of liver cirrhosis, biliary saccular dilation observed in imaging examinations, portal tracts fibrosis observed in liver biopsy, and the presence of PKHD1 mutation.

**Table 2:** Biological examination results

Parameters	Value	Unit	Reference Ranges
RBC	2.88*10^12^/L		4.0–5.5*10^12^/L
WBC	2.35*10^9^/L		4–10*10^9^/L
Hb	55.00 g/L	g/L	120–160
AST	16.6	U/L	0–40
ALT	13.7	U/L	0–40
AST/ALT	1.2		
TBiL	27.8	μmol/L	3.42–20.5
DBiL	9	μmol/L	0–6.84
ALB	37.4	g/L	35–55
ALP	70	U/L	0–150
LDH	127	U/L	109–245
CK	32	U/L	0–190
Cu	20.8	U/L	10–24.7
CER	320.7	mg/L	150–600
TBA	9.32	μmol/L	0–12
PA	122	mg/L	250–400
GR	48.6	U/L	33–73
SAA	92	mg/L	0–10
TC	2.05	mmol/L	2.8–5.7
HDL	0.58	mmol/L	1.16–1.55
LDL	1.04	mmol/L	0–3.61

A compound heterozygous mutation of the PKHD1 gene was identified through whole exome sequencing. The results indicate that both the patient and his affected sister have two heterozygous variants: a missense variant c.2507 T > C and a novel variant c.10156G > C. We searched for the c.2507 T > C mutation on the GnomAD website, and the results suggest that this mutation is likely to be pathogenic. This finding is consistent with several Chinese articles, which suggest that it is unique to Chinese individuals [34, 35]. However, there is no data available on the c.10156G > C mutation on the GnomAD website. We analyzed the clinical significance of this variant using polyPhen2, SIFT, MutationTaster, FATHMM, and PROVEAN. The details of this analysis are provided in Table 3. According to the American College of Medical Genetics (ACMG) standards and guidelines for variant interpretation, the c.10156G > C mutation is considered a likely pathogenic mutation in the PKHD1 gene for this family. Additionally, we investigated the minor allele frequency (MAF) of these two mutation sites in different populations. The details are presented in Table 4. The c.2507 T > C mutation was found to be a rare and low-frequency variant, with the MAFs of less than 5%. The c.10156G > C mutation has not been previously reported.

**Table 3:** Bioinformatics analysis of the mutations

Mutation	SIFT		PolyPhen2		MutationTaster		FATHMM		PROVEAN		InterVar
	Score	prediction	Score	prediction	Score	prediction	Score	prediction	Score	prediction	prediction
PKHD1 c.T2507C p.V836A	0	deleterious	0.861	possibly_damaging	0.667	Polymor phism	-2.58	deleterious	-2.77	deleterious	Pathogenic/ Likely pathogenic
PKHD1 c.G10156C p.V3386L	0.003	deleterious	0.83	possibly_damaging	0.998	disease_causing	-2.37	deleterious	-4.86	deleterious	Pathogenic/ Likely pathogenic

SIFT PREDICTION (cutoff = 0.05)—tolerated or damaging

PolyPhen Prediction Score benign ≤ 0.5; probably damaging (> 0.5)

MutationTaster Prediction polymorphism or disease causing

FATHMM Prediction (weighted) torlerated or damaging

PROVEAN Prediction (cutoff = -2.5)—deleterious or neutral

InterVar is the classification of variants into 'Benign', 'Likely benign', 'Uncertain significance', 'Likely pathogenic' and 'Pathogenic' based on ACMG/AMP 2015 guideline

**Table 4:** The minor allele frequency (MAF) in different populations

database	dbSNP	1000G_ ALL	1000G_ EAS	1000G_ SAS	ExAC_ ALL	ExAC_ EAS	ExAC_ SAS	gnomAD_ ESA	gnomAD_ SSA
PKHD1 c.T2507C p.V836A	rs199568593	0.0002	0.001	-	0.0001	0.0013	0	-	0.0006
PKHD1 c.G10156C p.V3386L	-	-	-	-	-	-	-	-	-

1000G 1000 Genomes Project

*EAS* East Asian, *SAS* South Asian, *ExAC* Exome Aggregation Consortium

*gnomAD* Genome Aggregation Database

The PKHD1 gene encodes the fibrocystin protein (FPC), which is located in the membrane of primary cilia. These primary cilia are involved in the formation of cysts in the kidney and liver [36]. Multiple reports have demonstrated that PKHD1 mutations are responsible for autosomal recessive polycystic kidney disease (ARPKD) and Caroli syndrome (CD) [37–39]. To date, over 900 PKHD1 mutations have been reported, with 60% being missense mutations and 40% being protein-truncated mutations [34] Ishiko et al. conducted a minigene assay and discovered that at least one non-truncating mutation of PKHD1 is necessary for perinatal survival [40]. However, published reports indicate that the same PKHD1 mutation can be associated with different clinical presentations [20, 41]. These findings highlight the complexity of the relationship between PKHD1 and CS. In summary, the identification of PKHD1 mutations can provide strong evidence for the diagnosis of CS. In this particular case, the PKHD1 mutation only resulted in hepatic presentations without renal disorder. This suggests that the compound heterozygous mutation could significantly contribute to the etiology of isolated Caroli syndrome. However, it is not the sole reason for the patient's clinical presentation. Additionally, environmental factors may also play a role in the pathogenesis of CS, although the mechanisms remain unclear [17, 34]. PKHD1 mutation is the most well-known cause of CS and can be easily confirmed through next-generation sequencing (NGS). However, there may be other yet undiscovered pathological factors that contribute to the disease. Given the sequence of events, the patient will require long-term follow-up to determine whether renal symptoms will develop.

In this case report, we successfully diagnosed a case of atypical CS, despite its limited mention in the literature. Supporting our findings, previous studies have also reported cases of atypical CS. For example, XiaoYM et al. documented a series of patients with CS who exhibited atypical symptoms and imaging manifestations [28]. Although our patient did not show the typical “central sign” in ultrasound and CT, multiple cysts were observed in the liver and kidney, which contrasted with our case report. Similarly, Acioli, M.L et al. described a case of atypical CS characterized by uncommon clinical manifestations, such as the absence of non-specific abdominal pain, cholestasis, or cholangitis. However, imaging examinations still proved effective in diagnosing CS [42]. Another study conducted by Tiotia, Rahul et al. reported a patient with atypical CS who, similar to our case, did not have renal lesions. However, this patient had typical intrahepatic manifestations, such as polycystic liver and fibrosis [43].

Generally, non-invasive imaging examinations, such as ultrasound, CT, and Magnetic Resonance Imaging (MRI), are sufficient for diagnosing CS. On the other hand, invasive procedures, including Magnetic resonance cholangiopancreatography (MRCP), Endoscopic Retrograde Cholangiopancreatography (ERCP), Percutaneous Transhepatic Cholangiography (PTC), and liver biopsy, are primarily used for differential diagnosis [27, 44, 45]. These procedures are only performed when patients present visible symptoms and seek medical attention. Consequently, individuals with a family history of liver cirrhosis or recurrent cholangitis should be made aware of the importance of PKHD1 gene sequencing by healthcare professionals, in order to increase the early detection rate during check-ups.

## Data Availability

Not applicable.

## Abbreviations

PKHD1: Polycystic kidney and hepatic diseases 1
CS: Caroli’s syndrome
CD: Caroli’s disease
CHF: Congenital hepatic fibrosis
ARPKD: Adult recessive polycystic kidney disease

## References

[1] Caroli J, Soupault R, Kossakowski J, Plocker L, Paradowska. Congenital polycystic dilation of the intrahepatic bile ducts; attempt at classification. La semaine des hopitaux : organe fonde par l'Association d'enseignement medical des hopitaux de Paris. 1958;34(8/2):488−95/sp. 13543375

[2] Mahajan R (2021). International journal of applied and basic medical research: decade-old journey of journal. Int J Appl Basic Med Res.

[3] Yao X, Ao W, Fang J, Mao G, Chen C, Yu L (2021). Imaging manifestations of Caroli disease with autosomal recessive polycystic kidney disease: a case report and literature review. BMC Pregnancy Childbirth.

[4] Williams SS, Cobo-Stark P, Hajarnis S, Aboudehen K, Shao X, Richardson JA (2014). Tissue-specific regulation of the mouse Pkhd1 (ARPKD) gene promoter. Am J Physiol Ren Physiol.

[5] Morales X, Jiménez-Hermida L, Pérez JM, Hernández-Cely G (2022). Role of cholangioscopy in a patient with hepatolithiasis and caroli disease. ACG case Rep J.

[6] Moslim MA, Gunasekaran G, Vogt D, Cruise M, Morris-Stiff G (2015). Surgical management of Caroli’s disease: single center experience and review of the literature. J Gastrointest Surg.

[7] Acevedo E, Laínez SS, Cáceres Cano PA, Vivar D (2020). Caroli’s syndrome: an early presentation. Cureus.

[8] Brancatelli G, Federle MP, Vilgrain V, Vullierme MP, Marin D, Lagalla R (2005). Fibropolycystic liver disease: CT and MR imaging findings. Radiographics.

[9] Jiang YX, Wang ZG, Hu B, et al. Medical Ultrasound Imaging. 2nd ed. People's Medical Publishing House; 2010.

[10] Bai RJ, Zhang XL, Meng FJ, et al. Diagnostic Medical Imaging. 4th ed. People's Medical Publishing House; 2010.

[11] Ru-Jia T, Zhen-Wen L, Hai-Bin SU, Xi HE, Da-Li Z, Xia Z (2013). The clinical features analysis of 48 cases of congenital hepatic fibrosis. Chin Hepatol.

[12] Ohmori S, Shiraki K, Sugimoto K, Sakai T, Fujikawa K, Wagayama H (2001). High expression of CD34-positive sinusoidal endothelial cells is a risk factor for hepatocellular carcinoma in patients with HCV-associated chronic liver diseases. Hum Pathol.

[13] Kleiner DE (2017). Drug-induced liver injury: the hepatic pathologist’s approach. Gastroenterol Clin N Am.

[14] Lefkowitch JH (2006). Special stains in diagnostic liver pathology. Semin Diagn Pathol.

[15] Ahmad A, Ahmad R (2014). Resveratrol mitigate structural changes and hepatic stellate cell activation in N’-nitrosodimethylamine-induced liver fibrosis via restraining oxidative damage. Chemico-Biol Interact.

[16] EASL Clinical Practice (2022). Guidelines on the management of cystic liver diseases. J Hepatol.

[17] Shi W, Yang AM (2021). Caroli disease: an update on pathogenesis. Chin Med J.

[18] Cnossen WR, Drenth JP (2014). Polycystic liver disease: an overview of pathogenesis, clinical manifestations and management. Orphanet J Rare Dis.

[19] Wills ES, Roepman R, Drenth JP (2014). Polycystic liver disease: ductal plate malformation and the primary cilium. Trends Mol Med.

[20] Courcet JB, Minello A, Prieur F, Morisse L, Phelip JM, Beurdeley A (2015). Compound heterozygous PKHD1 variants cause a wide spectrum of ductal plate malformations. Am J Med Genet Part A.

[21] Chung T, Rhee H, Shim HS, Yoo JE, Choi GH, Kim H (2022). Genetic, clinicopathological, and radiological features of intrahepatic cholangiocarcinoma with Ductal plate malformation pattern. Gut Liver.

[22] Pillai S, Center SA, McDonough SP, Demarco J, Pintar J, Henderson AK (2016). Ductal plate malformation in the liver of boxer dogs: clinical and histological features. Vet Pathol.

[23] Qiu LR, Xu RR, Tang JH, Zhou JH (2020). Possible PKHD1 hot-spot mutations related to early kidney function failure or hepatofibrosis in Chinese children with ARPKD: a retrospective single center cohort study and literature review. Curr Med Sci.

[24] Lasagni A, Cadamuro M, Morana G, Fabris L, Strazzabosco M (2021). Fibrocystic liver disease: novel concepts and translational perspectives. Translational Gastroenterol Hepatol.

[25] Fluhr TL, Tabatabaeifar M, Syring H, Göhring G, Schaefer F, Jung-Klawitter S (2021). Generation of an induced pluripotent stem cell line (DHMCi006-A) from a patient with autosomal recessive polycystic kidney disease (ARPKD) carrying a compound heterozygous missense mutation in the fibrocystin encoding PKHD1 gene. Stem cell Res.

[26] Li J, Liu LW, Luo J, Liu JX, Liu XJ, Zhu ZJ (2020). Clinicopathological features of Caroli disease/Caroli syndrome: an analysis of 21 cases. Zhonghua Yi Xue Za Zhi.

[27] Zhang DY, Ji ZF, Shen XZ, Liu HY, Pan BJ, Dong L (2012). Caroli’s disease: a report of 14 patients and review of the literature. J Dig Dis.

[28] Xiao YM, Peng TT, Liu YX (2021). A case of Caroli’s disease confirmed by pathology, atypical symptoms and images. Zhonghua Gan Zang Bing Za Zhi.

[29] Perricone G, Vanzulli A (2015). Education and imaging. Hepatology: central dot sign of Caroli syndrome. J Gastroenterol Hepatol.

[30] Schilsky ML (2017). Wilson Disease: diagnosis, treatment, and follow-up. Clin Liver Dis.

[31] Lucena-Valera A, Perez-Palacios D, Muñoz-Hernandez R, Romero-Gómez M, Ampuero J (2021). Wilson’s disease: revisiting an old friend. World J Hepatol.

[32] Sucher E, Sucher R, Gradistanac T, Brandacher G, Schneeberger S, Berg T (2019). Autoimmune hepatitis-immunologically triggered liver pathogenesis-diagnostic and therapeutic strategies. J Immunol Res.

[33] Tsuneyama K, Baba H, Morimoto Y, Tsunematsu T, Ogawa H (2017). Primary biliary cholangitis: its pathological characteristics and immunopathological mechanisms. J Med Investig.

[34] Hao X, Liu S, Dong Q, Zhang H, Zhao J, Su L (2014). Whole exome sequencing identifies recessive PKHD1 mutations in a Chinese twin family with Caroli disease. PLoS ONE.

[35] Yang XY, Zhu LP, Liu XQ, Zhang CY, Yao Y, Wu Y (2018). Genetic diagnosis of Caroli syndrome with autosomal recessive polycystic kidney disease: a case report and literature review. Beijing Da Xue Xue Bao Yi Xue Ban.

[36] Ma M (2021). Cilia and polycystic kidney disease. Semin Cell Dev Biol.

[37] Bergmann C, Senderek J, Windelen E, Küpper F, Middeldorf I, Schneider F (2005). Clinical consequences of PKHD1 mutations in 164 patients with autosomal-recessive polycystic kidney disease (ARPKD). Kidney Int.

[38] Zerres K, Mücher G, Bachner L, Deschennes G, Eggermann T, Kääriäinen H (1994). Mapping of the gene for autosomal recessive polycystic kidney disease (ARPKD) to chromosome 6p21-cen. Nat Genet.

[39] Guay-Woodford LM, Muecher G, Hopkins SD, Avner ED, Germino GG, Guillot AP (1995). The severe perinatal form of autosomal recessive polycystic kidney disease maps to chromosome 6p21.1-p12: implications for genetic counseling. Am J Hum Genet.

[40] Ishiko S, Morisada N, Kondo A, Nagai S, Aoto Y, Okada E (2022). Clinical features of autosomal recessive polycystic kidney disease in the Japanese population and analysis of splicing in PKHD1 gene for determination of phenotypes. Clin Exp Nephrol.

[41] Smolović B, Muhović D, Hodžić A, Bergant G, Peterlin B (2018). The role of Next Generation sequencing in the Differential diagnosis of Caroli’s syndrome. Balkan J Med Genetics: BJMG.

[42] Acioli ML, Costa LR, de Miranda Henriques MS (2014). Diffuse Caroli’s disease with atypical presentation: a case report. Annals Gastroenterol.

[43] Tiotia R, Sharma M, Narayani V, Singh S, Dewan V, Deswal S, et al. Atypical presentation of caroli’s syndrome: a case report. Indian J Case Rep. 2019;5(1):79-81.

[44] Madjov R, Chervenkov P, Madjova V, Balev B (2005). Caroli’s disease. Report of 5 cases and review of literature. Hepatogastroenterology.

[45] Pavone P, Laghi A, Catalano C, Passariello R (1996). Caroli’s disease: evaluation with MR Cholangiography. AJR Am J Roentgenol.

